# Frequency and factors associated with malnutrition among patients with achalasia and effect of pneumatic dilation

**DOI:** 10.1002/jgh3.12191

**Published:** 2019-05-14

**Authors:** Uday Chand Ghoshal, Prabhakar Kumar Thakur, Asha Misra

**Affiliations:** ^1^ Department of Gastroenterology Sanjay Gandhi Postgraduate Institute of Medical Sciences Lucknow India

**Keywords:** balloon dilation, Eckardt score, motor dysphagia, nutrition, treatment

## Abstract

**Background:**

Although achalasia patients are undernourished, studies are scant. We studied: (i) the frequency of malnutrition among these patients and (ii) the effect of pneumatic dilatation (PD) on malnutrition.

**Methods:**

A total of 70 adult achalasia patients and 70 healthy controls were evaluated through dietary recall, anthropometry, and biochemical parameters, and patients were reevaluated 6 months after PD.

**Results:**

Patients had lower intake of calories (median, interquartile range [IQR]: 1835.0 [1682.5–1915.0] *vs* 2071.5 [1950–2276.2] kcal/day, *P* < 0.001), protein (40.9 [36.3–42.2] *vs* 52.9 [45.7–62] g/day, *P* < 0.001), calcium (310 [192.5–392.4] *vs* 477.5 [350–560] mg/day, *P* < 0.001), and iron (6.7 [4.7–8.8] *vs* 10.1 [7.5–11.50] mg/day, *P* < 0.001) than controls. Patients had lower body mass index (BMI: 19.6 [16.6–22] *vs* 22.8 [19.5,29.1], *P* < 0.001), midarm circumference (MAMC; 20 [17.5–23] *vs* 24.1 [21.4–28.5], *P* < 0.001), biceps (BSFT; 3.1 [1.9–3.9] *vs* 5.5 [3.8–9.2] mm, *P* < 0.001), triceps’ skin fold thickness (TSFT; 5 [2.4–7] *vs* 7.8 [5.1–9.4] mm, *P* < 0.001), serum protein (7.2 ± 0.8 *vs* 7.6 ± 0.8 g/dL, *P* = 0.005), and albumin (4.0 [3.5–4.4] *vs* 4.1 [3.9–4.2] g/dL, *P* = 0.009). PD increased calories (1803 [950–2400] *vs* 2050 [1470–2950] kcal/day), protein intake (41.0 [22–70] *vs* 45.0 [37.5–80.0] gm/day), BMI (19.6 [12.8–30.0] *vs* 22.2[15.9–30.0] *P* = 0.001 for all), and MAMC (21 [14.1–32.0] *vs* 24.2 [15–32.0] cm, *P* = 0.03). Reduced intake was a determinant of malnutrition.

**Conclusions:**

Malnutrition is common in achalasia patients, and PD improved it.

## Graphical abstract

Malnutrition is common in achalasia, and pneumatic dilation improves nutritional status.

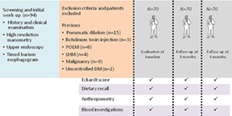

## Introduction

Achalasia is a specific esophageal motility disorder characterized by the absence of peristalsis and a defective swallow‐induced relaxation of the lower esophageal sphincter.^1^ It is caused by the progressive destruction and degeneration of neurons in the myenteric plexus. Typical symptoms of achalasia are dysphagia, regurgitation, retrosternal pain, and weight loss.^2^, ^3^ The disease is irreversible, and all current treatment options of achalasia are aimed at palliation of symptoms.^2^, ^4^ Dysphagia is the cardinal symptom of achalasia,^5^, ^6^ which results in inadequate dietary intake and the inability of the nutrients to reach the areas of the gut where these are absorbed. Hence, undernutrition is expected among patients with achalasia. Several series reported weight loss as a common symptom, varying between 30% and 90% among achalasia patients.^7^, ^8^, ^9^, ^10^, ^11^

Pneumatic dilatation (PD) is one of the most commonly used treatment modalities in patients with achalasia.^12^, ^13^ It results in symptomatic improvement in 80–90% patients^14^, ^15^; the response to treatment with PD may also last a long time.^16^ As successful PD results in the improvement of dysphagia and improves passage of foods to the sites where these are digested and absorbed, improvement in nutritional parameters following such an intervention is quite expected. However, there is no systematic study evaluating the frequency and factors associated with malnutrition in patients with achalasia and the effect of PD on it. We hypothesize that patients with achalasia would be malnourished, and successful PD would improve nutritional status. Accordingly, we undertook a prospective study with the following aims: (i) to estimate the prevalence of undernutrition in patients with achalasia compared to healthy subjects and (ii) to assess the change in nutritional status after PD.

## Methods

**Figure 1:**
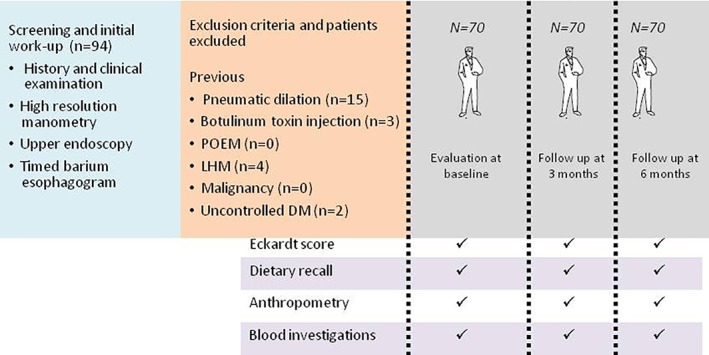
Outline of the study protocol, exclusion criteria, and the number screened and excluded and finally followed up. DM, diabetes mellitus; LHM, Laparoscopic Heller's myotomy; POEM, per‐oral endoscopic myotomy.

A total of 70 consecutive adult patients (≥18 years) with achalasia undergoing PD in a multilevel teaching hospital in northern India during a 1.5‐year period (July 2015 to January 2017) were recruited prospectively; 70 age‐ and gender‐matched healthy individuals (hospital staff members and healthy relatives of the patients volunteering to participate) were recruited as controls. Achalasia cardia was diagnosed by high‐resolution esophageal manometry using standard criteria. Exclusion criteria included previous endoscopic or surgical treatment and comorbid illnesses (e.g. active tuberculosis, uncontrolled diabetes mellitus, and malignancy). Clinical details were noted using a standard questionnaire. Both the patients and controls were evaluated for nutritional status using dietary survey, anthropometric, and biochemical parameters at baseline. In achalasia cardia patients, PD was performed using a Rigiflex dilator.^14^, ^15^ The success of PD was assessed using Eckardt score, esophageal manometry, and timed barium esophagogram after 4 weeks.^17^ Achalasia patients were followed for 6 months to assess changes in symptoms and nutritional parameters. The outline of the study protocol is shown schematically in Figure 1. The study protocol was approved by the Ethics Committee of the Institute (PGI/BE/570/2015, IEC CODE‐2015‐80‐DM‐85). Informed consent was obtained from each patient.

### *Clinical features*

The parameters evaluated included demographic and clinical details, including the Eckardt score at inclusion and on follow‐up. The Eckardt score was calculated as the sum of symptom scores of dysphagia, chest pain, regurgitation (0 = none, 1 = occasional, 2 = daily, 3 = at each meal), and weight loss (0 = none, 1 = <5 kg, 2 = 5–10 kg, 3 = >10 kg).^5^ The maximum possible score is 12, and a score < 3 after PD was considered to be indicative of clinical response. Details of esophageal manometry, esophagogastroduodenoscopy, and timed barium esophagogram were also recorded.

### *Nutritional assessment*

Dietary intake and anthropometric and laboratory parameters were evaluated to assess the nutritional status of participants. Malnutrition was defined using standard criteria.^18^ Briefly, individuals with a body mass index (BMI) < 18.5 kg/m^2^ were considered undernourished.

### *Assessment for dietary intake*

Participants were interviewed using the 72‐h recall method for the nature and amount (in terms of weight or household measures, e.g. standard cup set) of different food items such as cereals, pulses, vegetables, fats, milk, fruits, nuts, and other animal food products consumed.^19^ The nutrient contents of the dietary items were calculated using the nutritive values of Indian foods^20^ during each 24‐h period. Dietary intake was calculated by averaging an intake of 3 days.

### *Anthropometry*

BMI, waist–hip ratio (WHR), biceps (BSFT), triceps skin fold thickness (TSFT), and midarm circumference (MAMC) were recorded. BMI was calculated using the standard formula. Skin fold thickness was measured in the nondominant arm by a skin fold caliper using the standard technique. MAMC was measured in the nondominant arm at a midpoint between the tip of the acromion and the olecranon process using a measuring tape.^21^ Patients were described as undernourished if anthropometric measurements were lower compared to healthy subjects, as described previously.^21^

### *Laboratory parameters*

Hemoglobin (Hb) and serum total protein, albumin, calcium, and iron of all participants were measured at baseline.

### *Follow‐up*

Patients with achalasia were followed up at 3 and 6 months after PD. Dietary nutrient intake, clinical details, and anthropometric measurements were recorded at each visit.

### *Statistical analysis*

Nonparametric and parametric continuous and categorical data were presented as median and interquartile range (IQR), mean and standard deviation, and proportion, respectively. Various parameters were compared between cases and controls at baseline using the Mann–Whitney *U* test. Pre‐ and post PD comparisons among achalasia patients were conducted using Wilcoxon rank‐sum tests. More than two continuous variables were analyzed using one‐way analysis of variance and the post‐hoc Scheffe test. Data were analyzed using SPSS software (version 15.0 for Windows; SPSS, Chicago, IL, USA**)**. *P* values of <0.05 were considered significant.

## Results

### *Baseline characteristics*

Figure 1 shows the patients initially screened and the number excluded according to various exclusion criteria, finally including 70 patients with achalasia in the study. Table 1 shows the demographic and clinical parameters of patients with achalasia. Patients with achalasia were comparable to healthy subjects with regard to age (median 38, range 18–65 years *vs* 37, range 20–65) and gender (37/70, 52.8% male in each group). On high‐resolution manometry, of 70 patients with achalasia, 57 (81.4%) and 13 (18.6%) had type I and type II achalasia, respectively; no patient had type III achalasia. Prior to PD, median lower esophageal sphincter (LES) pressure was 32 mmHg (range 10–110), and integrated relaxation pressure (IRP) was 34.8 mmHg (range 12–79). The median duration of symptoms prior to diagnosis was 2 years (range 3 months to 20 years). Dysphagia was the most common symptom seen in 70 (100%); other symptoms were weight loss in 56 (80%), regurgitation in 42 (60%), chest pain in 29 (41.4%), difficulty in belching in 22 (31.4%), and hiccups in 4 (5.2%). All the patients had dysphagia both to solid and liquid foods before PD; dysphagia to both types of foods improved after serial PD in 68 patients, although 6 of these patients did report assistance of water while eating solid foods. The pretreatment median Eckardt score among patients with achalasia was 6 (range 3–12). Achalasia patients had a history of an average 5 kg (median, range 0–20 kg) weight loss at presentation.

**Table 1:** Clinical features of achalasia patients

Age (years)	38 (18–75)
Male, *n* (%)	37 (52)
Duration of symptoms (years)	2 (0.4–10)
Dysphagia, *n* (%)	70 (100%)
Weight loss, *n* (%)	56 (80%)
Regurgitation, *n* (%)	42 (60%)
Chest pain, *n* (%)	21 (30%)
Eckardt score	6 (2–12)
Weight loss (kg)	5 (0–20)

Continuous data are expressed as median and range and categorical data as number and percentages.

### *Nutritional assessment*

Daily intake of calories, proteins, calcium, and iron were lower in achalasia patients compared to healthy subjects (Table 2). Reduced dietary intake was seen in 62 (88%) achalasia patients but in none of the healthy controls. Patients with achalasia had lower BMI, BSFT, TSFT, and MAMC than healthy controls, although WHR was comparable (Table 2). Patients with achalasia had lower total serum protein and albumin than healthy controls. The levels of serum iron, calcium, and Hb, however, were comparable (Table 2).

**Table 2:** Nutritional parameters of achalasia patients at presentation and healthy control

	Patients with achalasia (*n* = 70)	Healthy controls (*n* = 70)	*P* values
Daily dietary intake			
Calorie (kcal/day)	1835.0 (1682.5–1915.0)	2071.5 (1950.0–2276.2)	<0.001
Protein intake (g/day)	40.9 (36.3–42.2)	52.9 (45.7–62.0)	<0.001
Iron intake (mg/day)	6.7 (4.7–8.8)	10.1 (7.5–11.5)	<0.001
Calcium intake (mg/day)	310.0 (192.5–392.4)	477.5 (350.0–560.0)	<0.001
Anthropometric measurements			
Body mass index (kg/m^2^)	19.6 (16.6–22.0)	22.8 (19.5–29.1)	<0.001
Waist–hip ratio	0.8 (0.8–0.9)	0.9 (0.8–0.9)	0.629
Midarm circumference (cm)	20.0 (17.5–23.0)	24.1 (21.4–28.5)	<0.001
Biceps skin fold thickness (mm)	3.1 (1.9–3.9)	5.5 (3.8–9.2)	<0.001
Triceps skin fold thickness (mm)	5.0 (2.4–7.0)	7.8 (5.1–9.4)	<0.001
Laboratory parameters			
Hemoglobin (g/dL)	12.0 (11.0–13.2)	12.3 (11.2–13.5)	0.281
Serum total protein (g/dL, mean (SD)	7.2 (0.8)	7.6 (0.8)	0.005
Serum albumin (g/dL)	4 (3.5–4.2)	4.1 (3.9–4.2)	0.009
Serum calcium (mg/dL)	8.8 (8.6–9.0)	8.9 (8.6–9.5)	0.073
Serum iron (mcg/dL)	45.0 (34.0–68.0)	51.0 (34.0–66.0)	0.894

All the continuous data are expressed as median and interquartile range except serum protein value.

### *Effect of PD*

The first session of PD resulted in improvement in dysphagia in 58 (82.8%) patients. The dysphagia score improved from 3 to 0 in 42 (60%) patients and from 2 to 0 in 28 (22.8%) after the first session of PD. In 12 (18%) patients, dysphagia improved transiently (*n* = 7) or persisted (*n* = 5) after the first session of PD. They underwent a second session of PD 4–12 weeks after the first session. Of 12 patients, 4 (33.3%) remained symptomatic after the second session of PD, and they underwent a third session. Two (2.8%) patients were nonresponsive to PD and remained symptomatic even after the third session of PD. These nonresponders to PD were young male patients (19 and 32 years old), and they underwent Heller's myotomy later on. No major complication of PD was observed in this study.

**Figure 2:**
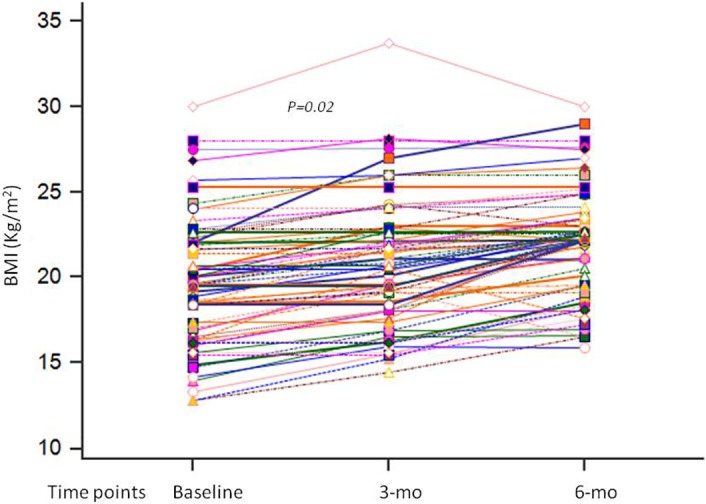
Body mass index (BMI, kg/m^2^) among patients with achalasia at baseline and during 3‐ and 6‐month follow‐up after pneumatic dilation.

Dietary intake of calories (*P* = 0.001), protein (*P* = 0.001), calcium (*P* = 0.001), and iron (*P* = 0.001) increased significantly 6 months after PD (Table 3). Although BMI (*P* = 0.001, Fig. 2) and MAMC (*P* = 0.03) increased, there was no significant change in BSFT (*P* = 0.32), TSFT (*P* = 0.82), and WHR (*P* = 0.9) 6 months after PD (Table 3). Reduced dietary intake, rather than any demographic and clinical parameter, was associated with malnutrition (Table 4).

**Table 3:** Nutritional parameters of achalasia patients before and 6 months after pneumatic dilation (PD)

Parameter	Baseline	6 months after PD	*P* value
BMI (kg/m^2^)	19.6 (12.8–30.0)	22.2 (15.9–30.0)	0.001
BSFT (mm)	3.1 (0.6–12.0)	3.2 (0.7–12.0)	0.32
TSFT (mm)	5.0 (0.4–20.5)	5.1 (0.4–20.5)	0.82
MAMC (cm)	21.0 (14.1–32.0)	24.2 (15.0–32.0)	0.03
WHR	0.8 (0.7–1.3)	0.9 (0.7–1.3)	0.9
Dietary intake			
kcal/day	1803.0 (950.0–2400.0)	2050.0 (1470.0–2950.0)	0.001
Protein (g/day)	41.0 (22.0–70.0)	45.0 (37.5–80.0)	0.001
Calcium (mg/day)	310.0 (123.0–1020.0)	358.0 (210.0–1150.0)	0.001
Iron (mg/day)	6.7 (3.0–15.0)	8.7 (3.4–15.0)	0.001

BMI, body mass index; BSFT, biceps skin fold thickness; MAMC, midarm circumference; TSFT, triceps skin fold thickness; WHR, waist–hip ratio.

**Table 4:** Factors associated with malnutrition among patients with achalasia cardia

	Undernutrition absent (*n* = 44)	Undernutrition present (*n* = 26)	*P* value
Age (years, median, IQR)	38.0 (28.0–42.0)	32.5 (21.0–40.5)	0.091
Gender (male)	56 (50%)	18 (64.3%)	0.253
Duration of symptoms (years, median, IQR)	2.0 (1.0–3.1)	2.0 (1.0–3.0)	0.264
Regurgitation	26 (59.1%)	16 (61.5%)	1
Eckardt score (mean, SD)	6.7 (1.7)	6.5 (1.4)	0.693
Daily calorie intake (kcal/day, median, IQR)	2000.0 (1877.5–2177.5)	1745.0 (1607.8–1850)	< 0.001
Daily protein intake (g/day, median, IQR)	45.8 (41.5–55.8)	38.1 (33.2–41.4)	< 0.001
Daily iron intake (mg/day, mean, SD)	8.8 (3.1)	6.6 (2.5)	< 0.001
Calcium intake (mg/day)	425.2 (317.5–520.0)	241.0 (184.6–367.6)	< 0.001
Hb (g/dL, median, IQR)	12.2 (11.2–13.5)	12 (10.4–13.2)	0.306
Protein (g/dL, median, IQR)	7.4 (6.9–7.8)	7.2 (7.0–7.8)	0.867
Albumin (g/dL, mean, SD)	4.0 (0.4)	3.9 (0.5)	0.115
Achalasia subtype (*n*, %)			
Type 1	36 (81.8%)	21 (80.8%)	1
Type 2	8 (18.2%)	5 (19.2%)	
LES pressure (mmHg, median, IQR)	31.5 (21.8–40.0)	24.6 (19.0–32.5)	0.243
Distal contractile integral (median, IQR)	135.0 (21.0–343.5)	45.5 (19.8–406.8)	1
Integrated relaxation pressure (mean, SD)	36.4 (15.8)	32.2 (14.8)	0.274

Hb, hemoglobin; IQR, interquartile range; LES, lower esophageal sphincter.

## Discussion

The current study shows that (i) dietary intake is low among untreated patients with achalasia compared with the healthy subjects, (ii) that this is commonly associated with undernutrition among these patients, and (iii) that PD is effective not only in relieving dysphagia but also undernutrition in patients with achalasia.

The present study showed that undernutrition is common in achalasia patients and is prevalent among both types I and II disease. Weight loss was seen in 80% of patients with achalasia. These patients had low BMI, MAMC, and skin fold thickness, although WHR was comparable. Weight loss and low BMI among these patients have also been reported in earlier studies.^5^, ^7^, ^8^, ^9^, ^10^, ^11^ This possibly occurs due to reduced dietary intake as was evidenced by the low intake of various nutrients among patients with achalasia compared to healthy controls. Reduced food intake might result from difficulty in swallowing, chest discomfort, and regurgitation; moreover, a reduced amount of food reaching the absorptive area of the gut might also contribute to undernutrition. A few case reports of achalasia patients presenting as anorexia nervosa have also been described.^22^, ^23^, ^24^, ^25^ These cases have been reported primarily in children and the adolescent age group. In our study, no patient presented with anorexia nervosa‐like symptoms, which might be because only adult patients were included in this study. Although our results showed macronutrient deficiency (low serum protein and albumin) among patients with achalasia, micronutrients (iron, Hb, and calcium) were comparable. This might result from the fact that inadequate intake and the reduced arrival of nutrients into the absorptive area of gut might not be enough to cause micronutrient deficiency; moreover, many micronutrients have enough body storage and physiological regulatory mechanisms to obviate deficiency in the face of reduced availability. Although undernutrition in patients with achalasia has been reported in earlier studies, this is perhaps the first systemically designed study to analyze this issue and evaluate the effects of PD on the nutritional outcome.

Our data showed that PD was not only effective in relieving dysphagia but also nutrient intake with consequent correction of undernutrition; although the results of this study are quite expected, no earlier study evaluated it systematically. The efficacy of PD as observed in this study is in accordance with the earlier literature, which reported a success rate up to 80–90%.^12^, ^14^, ^15^, ^16^, ^26^ We found that the success rate after the first PD was 82.8%, and 97.1% could be treated successfully with PD over 6 months. Only two (0.3%) patients failed to improve with PD and underwent Heller's myotomy. Surgical LES myotomy^15^, ^16^, ^27^ and peroral endoscopic myotomy (POEM)^28^, ^29^ are other treatment options with comparable success rates. However, PD is a more time‐tested treatment modality with an excellent success rate.^14^, ^30^, ^31^ It is also cheaper^32^, ^33^, ^34^ and is associated with fewer complications.^35^ However, it is possible that POEM and surgical myotomy might be more effective in improving nutrition as these are considered to be more effective in relieving dysphagia.^36^ Hence, studies comparing the efficacy of different methods of treatment for achalasia, such as PD, POEM, and surgical myotomy, in correcting malnutrition are needed. Severe malnutrition in patients with achalasia may have considerable therapeutic implications. As patients with severe malnutrition may not be candidates for surgical treatment of achalasia, they may not be subjected to PD as it has potential risks such as perforation that may require surgical treatment.^37^ Hence, patients may respond better to initial rescue therapies such as botulinum toxin injection followed by PD later, after their nutrition improves.

This study showed significant improvement in nutritional status after PD. Daily calories, protein, calcium, and iron intake increased significantly 6 months after PD. Patients gained an average of 5 kg weight on 6 months’ follow‐up. Hence, BMI and MAMC increased as expected. However, BSFT, TSFT, and WHR remained comparable, which might be related to the follow‐up period of only 6 months; we believe that a longer follow‐up period might have resulted in improvement in these parameters as well.

It is quite expected that the patients who have difficulty swallowing for a long time would show physical and laboratory findings of undernutrition, and these would improve once dysphagia resolved. Therefore, the results of this study could be anticipated without any doubt. However, a systematic study to assess malnutrition among patients with achalasia and controls and 6‐month follow‐up to assess its clinical and biochemical improvement in a reasonably large number of achalasia patients treated with sequential PD has not been reported earlier; therefore, documentation of such naturally expected results by a prospectively and systematically organized study is the original merit and strength of this study. However, this study has a few limitations, which include a relatively short period of follow‐up, use of the recall method for nutritional assessment, and study of only a few biochemical parameters to assess undernutrition. Early markers of malnutrition were not evaluated in this study. It is, however, expected that a longer follow‐up and assessment of more laboratory parameters of malnutrition would have strengthened our conclusions rather than refuting it as undernutrition is expected to improve over time after the patients’ dysphagia improve with PD. However, although our study showed a high success rate of dysphagia improvement following PD (97.1% after the third session), one may argue that dysphagia might recur over a longer duration of follow‐up. However, several studies showed that PD, when successful, leads to improvement over a reasonable period of follow‐up. Use of a few more biochemical markers of malnutrition (prealbumin, transferring, 24 h urinary creatinine) might have been useful to assess nutrition more completely. In spite of these limitations, we believe that dietary assessment, anthropometry, and a few biochemical parameters were enough to prove our hypothesis. Further studies can be conducted with longer follow up and including more nutritional parameters.

We conclude that undernutrition is common in patients with achalasia cardia and possibly results from reduced dietary intake. PD is associated with an increase in dietary intake and improvement in nutritional status.
